# Paclitaxel and carboplatin after disease progression on immune checkpoint inhibitor for patients with metastatic urothelial carcinoma

**DOI:** 10.37349/etat.2026.1002387

**Published:** 2026-07-29

**Authors:** Albert Jang, Miguel Muniz, Megan Spychalla, Timothy Burns, Amrit Singh, Elisabeth I. Heath, Brian A. Costello, Jacob J. Orme, J. Fernando Quevedo, Daniel S. Childs, Lance C. Pagliaro

**Affiliations:** IRCCS Istituto Romagnolo per lo Studio dei Tumori (IRST) "Dino Amadori", Italy; ^1^Division of Medical Oncology, Department of Oncology, Mayo Clinic, Rochester, MN 55905, USA; ^2^Department of Hematology and Oncology, Mayo Clinic Health System, Eau Claire, WI 54703, USA; ^3^Department of Hematology and Oncology, Mayo Clinic Health System, Mankato, MN 56001, USA

**Keywords:** urothelial carcinoma, carboplatin, paclitaxel, immune checkpoint inhibitor, rechallenge

## Abstract

**Aim:**

Despite advances with immune checkpoint inhibitor (ICI)-based regimens in the past decade, patients with metastatic urothelial cancer (mUC) face a poor prognosis with few approved options. Platinum-based chemotherapy with paclitaxel and carboplatin (TC) +/– ICI may be a feasible choice.

**Methods:**

We generated an IRB-approved, HIPAA-compliant retrospective database of patients at Mayo Clinic Cancer Center with mUC who started TC +/– ICI after disease progression on ICI from January 2018 through December 2024. Baseline demographics, clinicopathologic features, and treatment outcomes were extracted from the electronic health record. Kaplan-Meier method was used to calculate median duration of response (DOR), progression-free survival (PFS) and overall survival (OS).

**Results:**

There were 32 patients who fit inclusion criteria, including 31 patients who had disease progression on ICI in the metastatic setting and 1 patient who developed metastatic disease on adjuvant nivolumab. Patients received a median of 4 cycles (range 1–14) of TC over median 12 weeks (range 3–53). Overall, 81% of patients received TC concurrently with an ICI, and 28% continued maintenance ICI after TC discontinuation (at the treating oncologist’s discretion due to adequate response or toxicity). The best objective response rate was 41% and disease control rate was 75%, with median DOR of 4.8 months (IQR 2.7–10.4), median PFS of 4.6 months (IQR 3.3–10.2), and median OS of 9.4 months (IQR 6.3–15.9). Some patients in this series had very durable responses with PFS > 12 months and OS > 24 months. TC +/– ICI was overall well tolerated with expected type and severity of adverse events.

**Conclusions:**

Strategies to overcome ICI resistance are needed, and TC +/– ICI after disease progression on an ICI shows promising tolerability and efficacy in this setting for patients with mUC. Our series was notable for some patients having a durable response. Validation in larger cohorts is warranted.

## Introduction

Patients with metastatic urothelial cancer (mUC) have a poor prognosis despite a decade of significant treatment advances, notably regarding immune checkpoint inhibitors (ICIs) [1]. These include pembrolizumab as a single agent after progression on frontline platinum-based chemotherapy [2], or as frontline therapy for platinum-ineligible patients [3], and maintenance avelumab after treatment response to platinum-based chemotherapy [4]. More recently, the combination of enfortumab vedotin (EV) with pembrolizumab (referred to as EVP) based on EV-302 and the triplet of cisplatin, gemcitabine, and nivolumab based on CheckMate 901 have cemented ICIs as part of the frontline standard for most patients with mUC [5, 6]. Additionally, ICIs are now regulatory approved in the muscle-invasive bladder cancer (MIBC) setting with adjuvant nivolumab per CheckMate 274 and perioperative durvalumab per NIAGARA [7, 8].

For most patients with mUC treated with ICIs, eventual disease progression is inevitable. The median progression-free survival (PFS) for patients receiving EVP was 12.5 months [5], and for cisplatin, gemcitabine, and nivolumab, the median PFS was 7.9 months [6]. There are limited options remaining for patients who have disease progression on ICI-based therapy, especially for those who lack therapeutic targets such as *FGFR3* alterations or HER2 overexpression [9, 10].

The combination of paclitaxel and carboplatin (TC) is a commonly used chemotherapy regimen for many solid tumors. TC demonstrated modest efficacy and tolerability in the pre-ICI era in several phase 1 and phase 2 trials for mUC [11, 12]. Although TC is not an approved regimen and is not often used due to the success of cisplatin-based chemotherapy and ICI-based therapy, this may be appropriate in cases where patients still desire treatment and have a good performance status without remaining options. In this retrospective study, we evaluated the real-world use of TC +/– ICI in patients with disease progression while on an ICI for mUC at our institution.

## Materials and methods

This was a HIPAA-compliant study using patients who were evaluated at Mayo Clinic Cancer Center who received TC with or without concurrent ICI after disease progression on ICI from January 2018 through December 2024. The study was conducted in accordance with the Declaration of Helsinki and its subsequent amendments. This study was deemed exempt by the Mayo Clinic Institutional Review Board (IRB#: 20-011547), so individual patient consent was not required. Patients with a linked diagnosis of urothelial cancer as well as ICD-10 codes C64, C65, C66, C67, C68 were queried and filtered to have received TC alone or with one of the regulatory-approved ICIs of atezolizumab, avelumab, durvalumab, pembrolizumab, or nivolumab. All patients meeting criteria were then individually reviewed to determine whether their disease progressed while receiving ICI and were subsequently started on TC. This analysis included five patients from a previously reported series [13], and the data cutoff for follow-up was September 30, 2025.

Individual patient data were collected to identify demographic information and disease history, including gender, race, smoking history, primary disease site, and metastatic sites at the time of TC initiation, and subsequent treatments. Next-generation sequencing (NGS) data from either tumor tissue or liquid biopsy and PD-L1 status were collected if available. Disease response was assessed in accordance with RECIST 1.1 [14].

Descriptive statistics were used to summarize baseline characteristics. PFS was measured from the start of TC to the date of disease progression or the date of death, and the duration was censored at the date of last follow-up for those alive without disease progression. Overall survival (OS) was measured from the start of TC to the date of death and was censored at the date of last follow-up for survivors. The median duration of response (mDOR) was measured from the date of first response on imaging to the date of progression or death. The survivor distribution was estimated using Kaplan-Meier methods. Log-rank test was used to compare Kaplan-Meier curves for subset analyses.

## Results

### Patient characteristics

**Figure 1:**
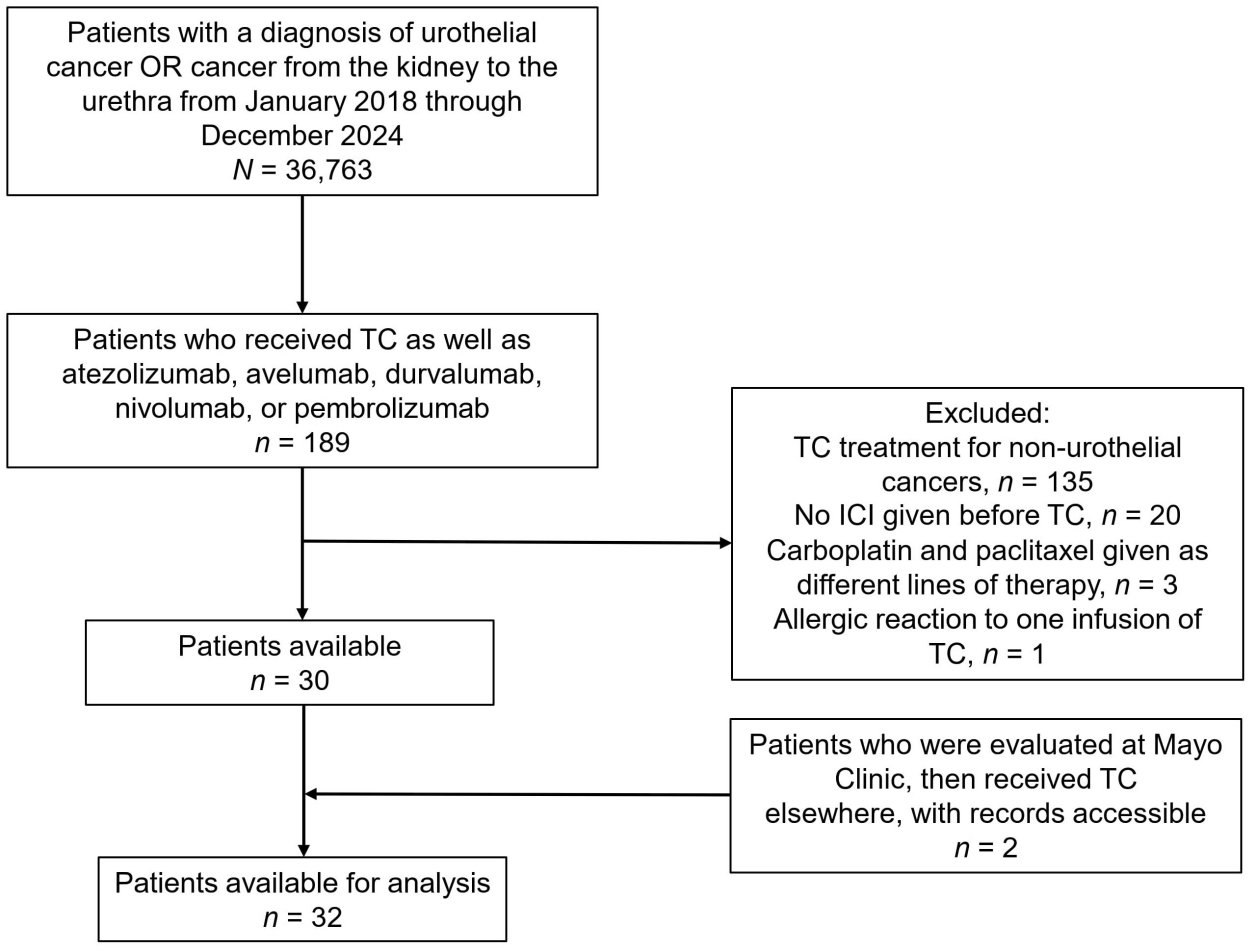
Consort diagram.

Between January 2018 and December 2024, there were 189 patients who received TC with exposure to ICI either during or before TC, and 30 patients with mUC received TC as the immediate next line of therapy after progression on an ICI (Figure 1). The most common reason for exclusion was TC treatment for non-urothelial cancer. There were two additional patients with ICI-refractory mUC who were initially evaluated and followed at our institution but received TC with a local oncologist. This included one patient who received two weekly doses of EV after progression on ICI while awaiting insurance approval of TC, before discontinuing EV due to poor tolerance requiring hospitalization and switching to TC. Altogether, our final analysis included 32 patients, of whom 31 had experienced progression on ICI in the metastatic setting and one had developed disease recurrence while on adjuvant nivolumab. Baseline cohort demographics are summarized in Table 1, and further details of systemic therapy prior to TC are provided in Table 2.

**Table 1:** Baseline characteristics.

**Baseline characteristics**	***n* (%) or median (range)**
**Total patients**	*n* = 32
**Gender**	
Male	25 (78%)
Female	7 (22%)
**Race**	
Caucasian	31 (97%)
Native American	1 (3%)
**Median age (year) at TC initiation (range)**	69 (35–85)
**Site of cancer**	
Upper tract	11 (34%)
Kidney	8 (25%)
Ureter	3 (9%)
Lower tract	20 (63%)
Bladder	19 (59%)
Urethra	1 (3%)
Unknown	1 (3%)
**Predominant histology**	
Urothelial	32 (100%)
**Variant histology component**	7 (22%)
Squamous	4 (13%)
Plasmacytoid	1 (3%)
Neuroendocrine	1 (3%)
Sarcomatoid	1 (3%)
**Sites of metastases at TC initiation**	
Baseline liver metastases	8 (25%)
Baseline lung metastases	14 (44%)
Baseline bone metastases	10 (31%)
Baseline LN metastases	20 (63%)
Baseline LN only metastases	6 (19%)
Baseline CNS metastases	0 (0%)
**Prior surgical resection of primary tumor**	13 (41%)
Prior cystectomy	8 (25%)
Prior nephroureterectomy	4 (13%)
Prior ureterectomy	1 (3%)
Prior neoadjuvant chemotherapy	6 (19%)
Neoadjuvant cisplatin/gemcitabine	4 (13%)
Neoadjuvant ddMVAC	2 (6%)
Prior adjuvant therapy	3 (9%)
Adjuvant ddMVAC	1 (3%)
Adjuvant cisplatin/gemcitabine	1 (3%)
Adjuvant nivolumab	1 (3%)
**Baseline documented hearing loss**	8 (25%)
**Baseline documented peripheral neuropathy**	12 (38%)
**Patients with solitary kidney at TC initiation**	4 (13%)
**ECOG performance status**	
0	16 (50%)
1	11 (34%)
2	4 (13%)
3	1 (3%)
**Median BMI (kg/m^2^) (range)**	25.1 (15.3–36.6)
**Median estimated GFR (mL/min/1.73m^2^) (range)**	57 (31–90+)
**Median Hgb (g/dL) (range)**	11.9 (8.2–15.8)
**Median Plt (K/µL) (range)**	265 (54–888)
**Median WBC (K/µL) (range)**	7.9 (3.7–20.7)
**Median ANC (K/µL) (range)**	5.2 (1.5–18.4)

TC: paclitaxel and carboplatin; LN: lymph node; CNS: central nervous system; ddMVAC: dose-dense methotrexate, vinblastine, doxorubicin, cisplatin; ECOG: Eastern Cooperative Oncology Group; BMI: body mass index; GFR: glomerular filtration rate; Hgb: hemoglobin; Plt: platelet; WBC: white blood cell; ANC: absolute neutrophil count.

**Table 2:** Systemic therapy prior to TC for 32 patients.

**Systemic therapy**	***n* (%) or median (range)**
**Prior frontline systemic therapy**	31 (97%)
Platinum-based chemotherapy	12 (38%)
Non-platinum-based chemotherapy	1 (3%)
ICI monotherapy	14 (44%)
ICI-based clinical trial	1 (3%)
Erdafitinib	1 (3%)
Enfortumab vedotin and pembrolizumab	2 (6%)
**ICI prior to starting TC**	32 (100%)
Pembrolizumab	25 (78%)
Avelumab	3 (9%)
Nivolumab	2 (6%)
Atezolizumab	2 (6%)
**Median number of ICI cycles (range)**	6 (1–22)
**Median weeks of ICI (range)**	16 (3–225)
**Number of lines of systemic therapy in the metastatic setting prior to starting TC**	
0	1 (3%)*
1	19 (59%)
2	11 (34%)
3+	1 (3%)

TC: paclitaxel and carboplatin; ICI: immune checkpoint inhibitor. *: This patient received adjuvant nivolumab, and therefore TC was a first-line metastatic regimen.

### Treatment outcomes

**Figure 2:**
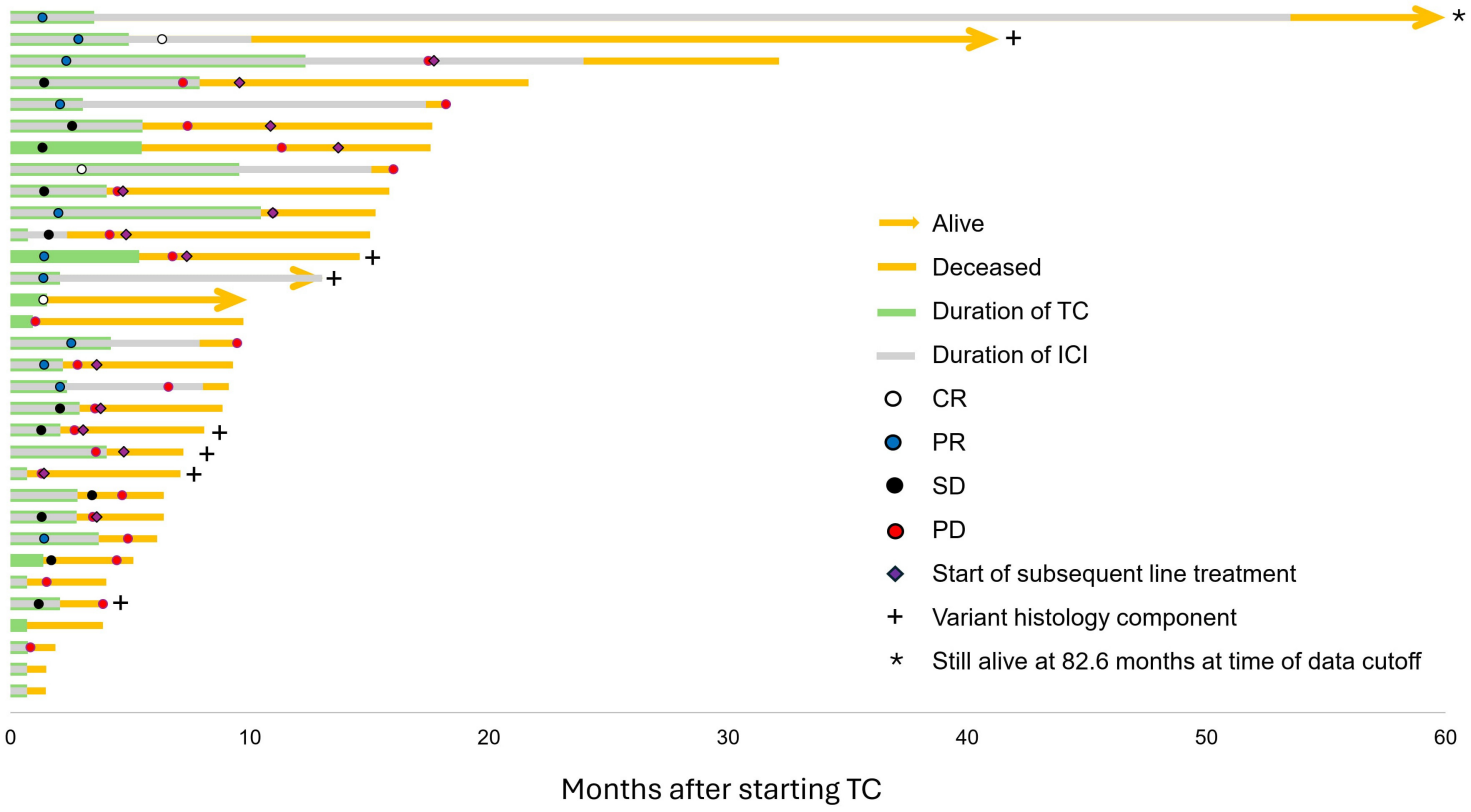
**Swimmer plot.** CR: complete response; ICI: immune checkpoint inhibitor; PD: progressive disease; PR: partial response; SD: stable disease; TC: paclitaxel and carboplatin.

Patients received a median of 4 cycles (range 1–14) over a median 12 weeks (range 3–53). Six patients (19%) received weekly TC, with a median starting dose of 45 mg/m^2^ (range 40–60) for paclitaxel and AUC 1.75 mg/mL·min (range 1.5–2) for carboplatin. Twenty-six patients (81%) received TC every 3 weeks, with a median starting dose of 162.5 mg/m^2^ (range 120–225) for paclitaxel and AUC 5 mg/mL·min (range 4–6) for carboplatin. Four patients required dose reduction of carboplatin and 6 patients required dose reduction of paclitaxel. There were 26 patients who also received ICI in conjunction with TC, with 25 receiving pembrolizumab and one receiving atezolizumab, with a median of 5.5 doses of ICI administered over a median of 16.6 weeks (range 3–230). Nine patients continued pembrolizumab after TC was discontinued due to the treating oncologist’s preference of either sufficient response or lack of tolerance. Further treatment details regarding TC +/– ICI are provided in Table 3. A swimmer plot for all 32 patients is shown in Figure 2.

**Table 3:** Information regarding TC +/– ICI for 32 patients.

**Information**	***n* (%) or median (range)**
**Median number of weeks on TC (range)**	12 (3–53)
Median number of cycles (range)	4 (1–14)
**Dosing every 3 weeks**	26 (81%)
**Dosing weekly**	6 (19%)
**Dose reduction of carboplatin required**	4 (13%)
**Dose reduction of paclitaxel required**	6 (19%)
**Paclitaxel held/discontinued due to peripheral neuropathy**	6 (19%)
**ICI added to TC**	26 (81%)
**Maintenance ICI after TC discontinuation**	9 (28%)
Median number of weeks of ICI after starting TC (range)	16.6 (3–230)
Median number of doses of ICI after starting TC (range)	5.5 (1–74)

TC: paclitaxel and carboplatin; ICI: immune checkpoint inhibitor.

**Figure 3:**
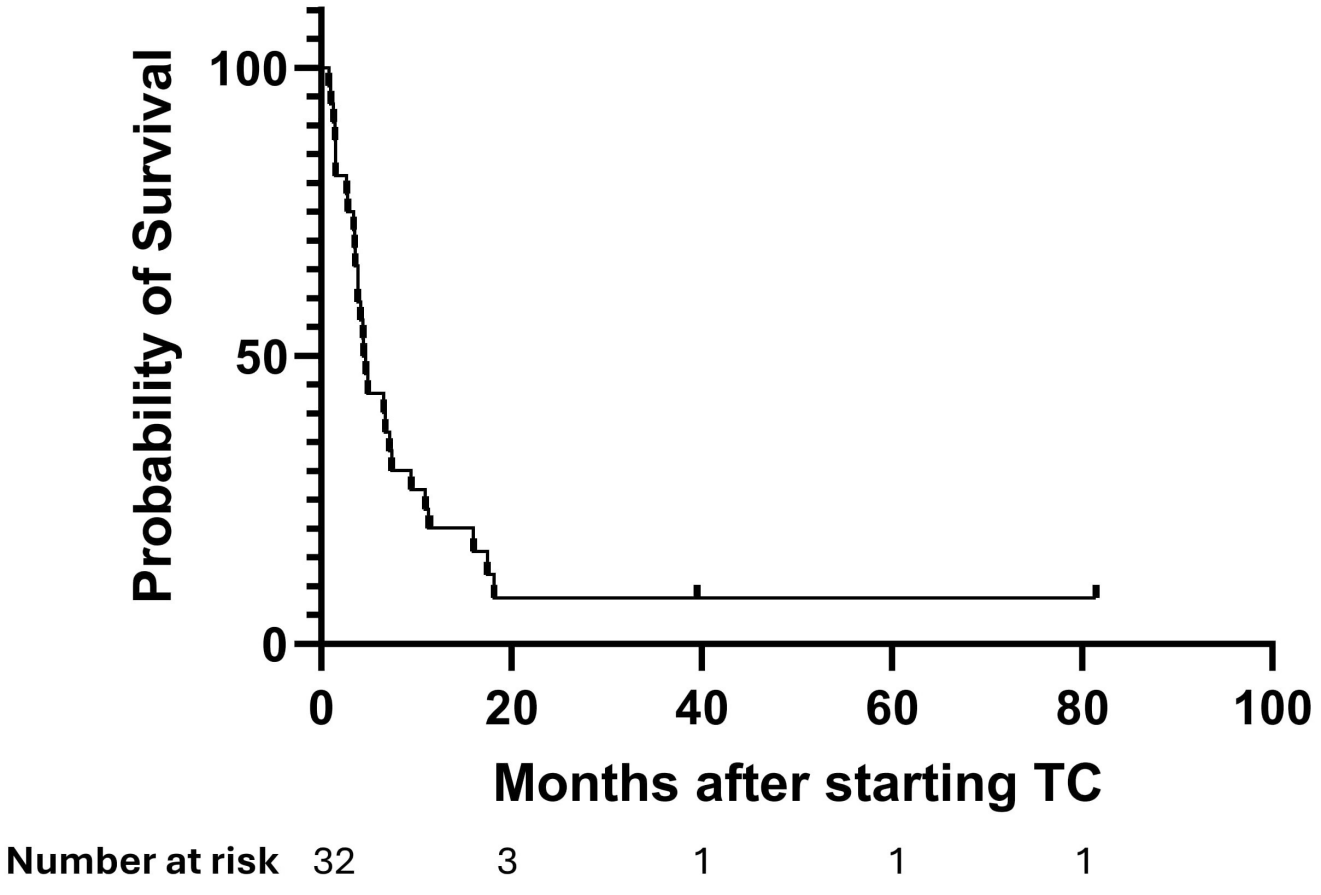
**Progression-free survival of the cohort.** The median progression-free survival was 4.6 months (IQR 3.3–10.2).

**Figure 4:**
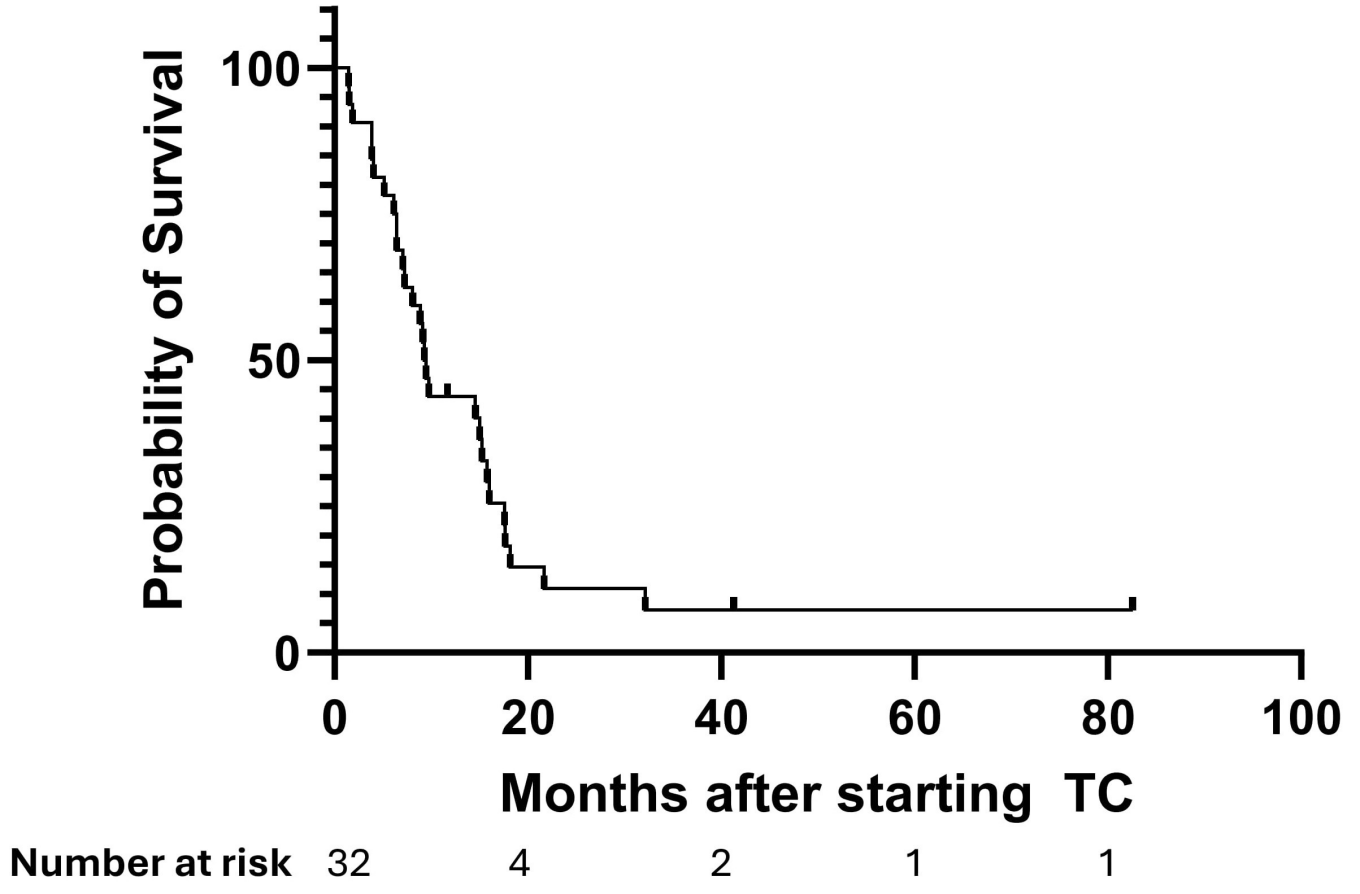
**Overall survival of the cohort.** The median overall survival was 9.4 months (IQR 6.3–15.9), and 4 patients were still alive at last follow-up.

Among the 32 patients, 3 (9%) achieved a complete response, 10 (31%) had a partial response (PR), 11 (34%) had stable disease (SD), and 8 (25%) had progressive disease (PD) as their best overall response. Notably, among the 8 PD cases, 5 patients had disease progression at the first restaging scan and 3 had already developed clinical progression before the first restaging imaging. This yielded an objective response rate (ORR) of 41% and a disease control rate (DCR) of 75%. The mDOR was 4.8 months (IQR 2.7–10.4), the median PFS was 4.6 months (IQR 3.3–10.2) (Figure 3), and the median OS was 9.4 months (IQR 6.3–15.9) (Figure 4). There were a few select patients with very durable response of PFS >12 months (*n* = 6) and OS >24 months (*n* = 3) after starting TC. When stratifying survival outcomes based on line of treatment in the metastatic setting (TC as first or second line versus third or greater), there was no significant difference in outcomes. When TC was used as first/second line treatment (*n* = 20), the median PFS was 4.3 months and the median OS was 9.5 months. When TC was used as third or greater line treatment (*n* = 12), the median PFS was 6.9 months and the median OS was 9.3 months. When stratifying survival outcomes based on prior surgery to remove the primary tumor (*n* = 13) with those who did not have surgery (*n* = 19), there was also no significant difference in outcomes. With prior surgery, the median PFS was 4.5 months, and the median OS was 6.4 months. With no prior surgery, the median PFS was 6.6 months, and the median OS was 9.5 months. When stratifying outcomes for patients with liver metastases (*n* = 8) compared to those without liver metastases (*n* = 24) when starting TC, there were no significant differences, although the median values were longer for patients without liver metastases. For patients with liver metastases, the median PFS was 3.9 months, and the median OS was 6.6 months. For patients without liver metastases, the median PFS was 5.6 months, and the median OS was 12.2 months. At time of data cutoff, 4 patients remain alive, on either observation (*n* = 3) or maintenance pembrolizumab (*n* = 1).

### Potential factors associated with outcomes

There were 20 patients with NGS data from either tumor tissue or liquid biopsy prior to starting TC. No specific alterations were associated with either treatment response or resistance to TC. The most common alterations noted were *TP53* and *TERT* promoter. Of note, two patients had *FGFR3* alterations—one patient had already received erdafitinib before receiving TC, and the other patient passed away before the regulatory approval of erdafitinib. There were PD-L1 CPS data available for four patients, with scores ranging from 0–60 and not associated with predictability for outcomes.

### Side effects of treatment

Overall, TC +/– ICI was well tolerated, with adverse events consistent with known profiles of carboplatin, paclitaxel, and immunotherapy. Four patients required dose reduction of carboplatin, and six patients required dose reduction of paclitaxel. The most common adverse events included development and/or worsening of documented peripheral neuropathy in 50% of patients (*n* = 16, including 15 of whom received at least 2 cycles), and six patients discontinued paclitaxel due to significant peripheral neuropathy. Of the 12 patients with already documented pre-existing peripheral neuropathy, four patients had documented worsening neuropathy, including two patients who required treatment discontinuation. Other common adverse events requiring dose reduction or delays included neutropenia, anemia requiring red blood cell transfusion, and/or thrombocytopenia (*n* = 7). Other documented side effects not leading to dose reduction or delays included mild infusion reactions, myalgias, and arthralgias. There were three cases of suspected immune-related adverse events (irAEs), generally limited to mild dermatitis and diarrhea, although one case was suspected of being a grade 5 neurological irAE.

### Next-line treatments

Fourteen patients (44%) had a subsequent line of treatment upon progression of disease, with the median time to next treatment after TC initiation of 4.8 months (range 1.4–17.7). One patient was rechallenged with paclitaxel, and two patients were rechallenged with TC. Other regimens included sacituzumab govitecan (*n* = 3), EV (*n* = 2), EVP (*n* = 2), selpercatinib (*n* = 1), carboplatin and gemcitabine (*n* = 1), cisplatin and gemcitabine (*n* = 1), and cisplatin, gemcitabine, and ifosfamide (*n* = 1). Eleven patients went onto a second line of treatment after TC, five patients received a third line, and one patient received a fourth line.

## Discussion

To our knowledge, this is the largest real-world cohort of TC +/– ICI after disease progression on ICI for patients with mUC. The regimen showed encouraging efficacy and a good safety profile, with an ORR and a DCR of 41% and 75%, respectively, including a few exceptional and durable responses in heavily pretreated patients. We demonstrate that patients with mUC with disease progression on ICI, who can tolerate another line of therapy but are ineligible for clinical trials or other approved treatments, may be good candidates for TC +/– ICI. Although there were some patients who had a durable response to TC +/– ICI, this cohort was too small to determine predictive markers of response.

One retrospective study characterized a similar patient cohort, which evaluated seven patients who had disease progression on both platinum-based chemotherapy and an ICI before receiving TC as salvage therapy [15]. That small series reported an ORR of 28.6% (2 PR, 4 SD, and 1 PD) as best responses, a median PFS of 3.4 months, and a median OS of 10.9 months. Five of seven patients developed grade ≥ 3 adverse events on TC. Overall, these outcomes are in the same range as our results (ORR 41%, median PFS 4.6 months, median OS 9.4 months), indicating broadly comparable efficacy. Notably, none of the patients in the prior study received immunotherapy beyond their initial ICI failure, whereas most patients in our cohort continued or resumed an ICI alongside TC.

TC can be given once every 3 weeks or weekly at lower doses. The comparison of TC every 3 weeks versus weekly in terms of PFS and OS has been evaluated both prospectively and retrospectively in ovarian cancer, with no significant differences in outcomes [16–18]. Weekly dosing may, however, be better tolerated in frail patients due to closer monitoring and easier dose adjustments [19]. In our cohort, the six patients who received weekly TC over a median of 14.6 weeks (range 3–34 weeks) had 2 PR, 1 SD, and 3 PD as best responses, achieving a similar spectrum of outcomes to those treated with the standard three-week schedule.

EVP is now widely considered the standard of care for mUC [5]. Unfortunately, some patients have significant side effects, with the three most common grade ≥ 3 adverse events of EVP being maculopapular rash, hyperglycemia, and neutropenia in the EV-302 trial. In our cohort, two patients had received frontline EVP. One patient discontinued EVP after one cycle due to hospice enrollment, then came off hospice and continued pembrolizumab monotherapy before developing PD. The patient then received 4 cycles of TC plus pembrolizumab (TCP) and continued to have PR on maintenance pembrolizumab at time of data cutoff. Another patient was intolerant of the first cycle of EVP, with hospital admission for a full body rash and disseminated intravascular coagulation thought to be secondary to refractory disease. EVP was discontinued and TCP was then administered successfully over 17 weeks before further disease progression. Additionally, a third patient was unable to tolerate one cycle of EV monotherapy after the disease progressed on pembrolizumab monotherapy, requiring hospitalization for severe hyperglycemia. This patient was switched to TCP, which was given for 34 weeks duration with SD as the best response. These examples illustrate a potential role for TCP when patients are unable to tolerate EV or when single-agent immunotherapy is insufficient. It is noteworthy that patients who develop peripheral neuropathy from EV may be at increased risk of worsening this neuropathy with TC. Given that EVP has become a standard of care combination in the frontline setting, patients receiving TC after EV must be monitored carefully for any worsening of neuropathy.

There are plausible biological reasons why adding chemotherapy could restore responsiveness to immunotherapy in some patients. Chemotherapy is thought to synergize with ICIs by depleting immunosuppressive cells, stimulating T-cell activity, and increasing the release of tumor antigens [20]. In our cohort, certain patients experienced tumor shrinkage when TC was added to ongoing pembrolizumab, and notably their responses were maintained for an extended period on maintenance ICI even after TC was stopped. This pattern suggests that TC might have helped overcome ICI resistance, effectively re-sensitizing the tumor to immunotherapy in those cases. A similar phenomenon was reported in a case series of five patients with various solid tumors who, despite disease progression on frontline chemotherapy and second-line ICI, later achieved significant radiographic responses with third-line chemotherapy [21]. Identifying which patients are likely to benefit from such chemoimmunotherapy re-sensitization is an important next step. Although NGS and PD-L1 immunohistochemistry were available for some patients, no definitive conclusions to predict outcomes using this information could be determined.

ICI now has an established role in the localized MIBC setting with perioperative durvalumab and adjuvant nivolumab. Perioperative EVP is now establishing a role in MIBC as well with the positive results seen in KEYNOTE-905/EV-303 and KEYNOTE-B15/EV-304 [22, 23]. As currently approved systemic treatments shift to being administered in the localized setting and cancer cells acquire resistance, developing strategies to overcome resistance to ICI becomes even more imperative. Our findings contribute to this evolving treatment landscape by suggesting one such strategy when standard options have been exhausted.

This study has several limitations inherent to its retrospective nature. First, not all treatment-related adverse events may have been captured or graded consistently according to the Common Terminology Criteria for Adverse Events in the medical record, so the true toxicity profile of TC could be underreported. Second, the study lacked a comparison between TC alone and TC plus ICI, so the added value of continuing ICI therapy in this context remains uncertain. Third, our cohort was limited to a single academic center and was predominantly Caucasian, which may limit the generalizability of our findings to more diverse populations. Additionally, NGS of tumor tissue or blood and PD-L1 staining were not routinely done or available for review in all patients given that these were not standard for all patients, so conclusions could not be drawn. Further, our cohort was subject to a potential selection bias, as patients had to be fit enough to receive further chemotherapy, which may exclude frail patients who after ICI may have gone to hospice or best supportive care instead of TC. We acknowledge that many patients in our cohort were treated in an era with fewer regulatory-approved options, given the rapid pace of advancement in mUC in the past few years. Nevertheless, this study provides real-world evidence for patients to have a viable option if there are no other regulatory-approved treatments or clinical trials available, with the potential to induce durable responses in some situations.

This study highlights the efficacy of TC +/– ICI for patients with mUC after disease progression on ICI. Although significant advances have been made in the past decade in treating mUC, disease relapse still occurs. The option of TC +/– ICI after disease progression on chemotherapy and ICI should be considered in patients not eligible for a clinical trial and without any targetable alterations, as some patients may achieve a durable long-lasting response despite prior treatment. Predicting this subset of patients remains unknown. Given the treatment revolution of EVP as frontline systemic treatment, patients who have PD on EVP or discontinue EV due to intolerance might have the chance to benefit from TC or TCP if they are able to tolerate further systemic treatment.

## Abbreviations

AUC: area under the curve
CPS: combined positive score
DCR: disease control rate
DOR: duration of response
EV: enfortumab vedotin
EVP: enfortumab vedotin and pembrolizumab
ICI: immune checkpoint inhibitor
IQR: interquartile range
irAEs: immune-related adverse events
mDOR: median duration of response
MIBC: muscle-invasive bladder cancer
mOS: median overall survival
mPFS: median progression-free survival
mUC: metastatic urothelial cancer
NGS: next-generation sequencing
ORR: objective response rate
OS: overall survival
PD: progressive disease
PFS: progression-free survival
PR: partial response
SD: stable disease
TC: paclitaxel and carboplatin
TCP: paclitaxel, carboplatin, and pembrolizumab

## Declarations

### Availability of data and materials

The raw data supporting the conclusions of this manuscript will be made available by the authors, without undue reservation, to any qualified researcher.

### Copyright

© The Author(s) 2026.
